# Massive Chondrolysis and Joint Destruction after Artificial Anterior Cruciate Ligament Repair

**DOI:** 10.1155/2021/6634935

**Published:** 2021-05-26

**Authors:** Jack Carlson, Olivia Fox, Peter Kilby

**Affiliations:** ^1^Department of Orthopaedics, Bathurst Hospital, Bathurst, NSW 2795, Australia; ^2^University of Notre Dame, School of Medicine, Sydney, NSW, Australia

## Abstract

The Ligament Augmentation Reconstruction System (LARS) is an artificial ligament made of polyethylene terephthalate (PET) used for anterior cruciate ligament (ACL) reconstruction in Australia. Poor results with previous generations of synthetic grafts causing synovitis, graft failure, and premature osteoarthritis have encouraged the production of the newer LARS ligament with good results. We present a case of massive chondrolysis and joint destruction after LARS implantation requiring total knee replacement in a 23-year-old male. This case documents a rare and severe complication to the LARS ligament as caution for the implementation of this device in young athlete.

## 1. Summary of Events

**Figure 1:**
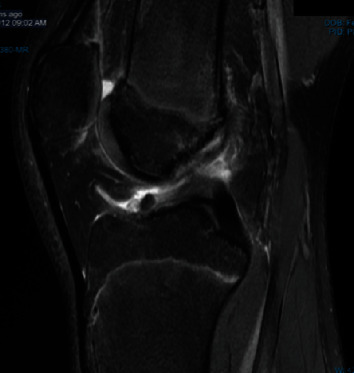
MRI demonstrating initial ACL rupture in 2012.

Mr. BP, a 16-year-old male, had a soccer injury in 2012 sustaining a left anterior cruciate ligament (ACL) and lateral meniscal tear (Figure 1). He subsequently had an ACL reconstruction performed by an orthopaedic surgeon (BM) who used the ipsilateral hamstring tendons for the ACL graft and repaired the lateral meniscus. A year later in 2014, Mr. BP sustained another injury to his left knee when dismounting from a dirt bike resulting in an intrasubstance ACL graft rupture and medial meniscus tear. He underwent a revision ACL reconstruction performed by another orthopaedic surgeon (EJ) using the contralateral (right) double hamstring tendons in addition to a Ligament Augmentation and Reconstruction System (LARS). The left medial meniscus was repaired with the omnispan meniscal repair system, and the frayed free edge was debrided conservatively to a stable margin.

In 2015, the patient sustained another knee injury with an MRI demonstrating intact ligaments, synovitis, and debris. The left medial meniscus remnant showed slight irregularities, and there were signs of early chondral wear in the medial compartment. The lateral meniscus showed a small radial tear that has been sutured previously with fraying and irregularity of the free edge.

Approximately 18 months since the revision ACL reconstruction, in 2016, Mr. AB was referred to a third orthopaedic surgeon (LH) regarding ongoing atraumatic pain and swelling in his left knee. The limb had a range of motion was from 0 to 130°. He had a grade II laxity with end point of his ACL. Both pivot shift sign and McMurray's test were negative, and there was no hip irritability. Blood tests such as erythrocyte sedimentation rate (ESR) and C-reactive protein (CRP) were normal.

Four (4) months later, Mr. AB underwent a left knee arthroscopy finding a very large haemarthrosis. The patellofemoral joint showed softening of the cartilage. The ACL graft was intact with no evidence of fraying. The LARS was visible in spots; however, there was marked synovitis in the notch, which was surgically debrided. The medial and lateral compartments showed softening of the cartilage with synovitis above and below the meniscus. The meniscus was intact in both compartments. Synovitis was abundant throughout the knee with the suprapatellar notch extending superiorly above the length of the scope. Coagulation studies excluded platelet and coagulation disorders as a systemic explanation for the large haemarthrosis.

Two weeks later, in 2016, Mr. BP represented with another episode of atraumatic pain and swelling within the knee after an initial period of improvement postarthroscopy. Mr. AB was presumed to have an arteriovenous (AV) malformation causing the recurrent bleeds into the knee and was referred for a Magnetic Resonance Angiography (MRA). In 2017, Mr. BP returned to EJ, in which a second arthroscopy was performed. This visualised significant haemarthrosis and florid synovitis, tricompartmental chondral fragmentation, and delamination. The medial meniscus was largely absent as it was at the time of the last surgery secondary to the previous trauma. The ACL graft was intact; however, one bundle was seen in the anterior aspect of the graft. This was isolated, resected, and removed as a precaution. The remaining graft was left intact. There was no significant synovitis over this area; however, there was synovitis in the rest of the pouch consistent with a pigmented villonodular synovitis (PVNS) type pattern. There was a degree of irritation but no obvious single bleeding vessel.

One month later, Mr. BP represented to the Emergency Department with another episode of atraumatic left knee pain and swelling. A referral was made for vascular embolisation at a tertiary centre. Angiography of the left knee showed hypervascularity around the knee due to synovial hypertrophy. At least 70% of the knee was devascularised during the procedure. Mr. BP was reviewed by another orthopaedic surgeon in Sydney (RB) two weeks later and was noted to be clinically improved and pain-free with only a small effusion in the left knee.

**Figure 2:**
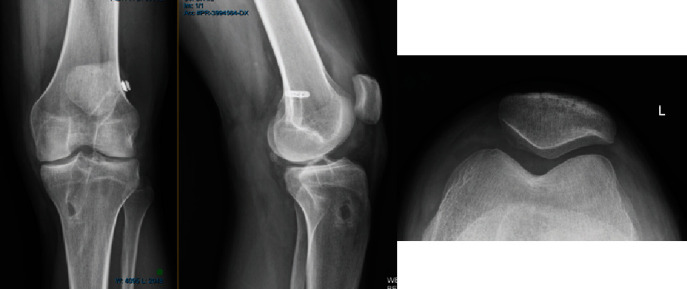
Radiograph in 2016 of BP's left knee.

The following year, Mr. BP represented with a locking sensation in his left knee, and he was then reviewed by another orthopaedic surgeon (PK). This MRI showed an intact ACL graft and features of haemosiderin staining and loss of synovium consistent with either recurrent haemarthosis or PVNS. There was further medial and lateral compartmental destruction with bone-on-bone appearances (Figure 2). The patellofemoral compartment showed early chondral damage, and both menisci were noted to be severely eroded and blunted. Conservative management was advised.

**Figure 3:**
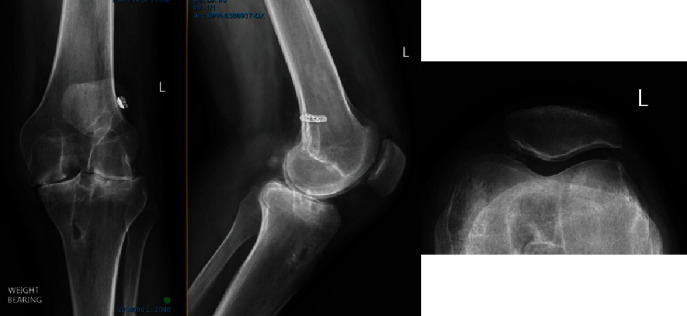
Radiograph in 2019 of BP's left knee. Note the significant interval change from 2016 images.

In March 2019, 6 years from the initial injury, Mr. BP (23 years) represented to PK for worsening pain and clunking with movement of his left knee. The pain was now both throughout the day and night and worse with work and not relieved with simple analgesia. On examination, the left quadriceps muscle was severely wasted, and the left knee had a positive pivot shift test and mild lateral ligament pseudo-laxity. A radiograph at the time showed severe chondrolysis with bone-on-bone disease in a pattern across all three compartments (Figure 3).

**Figure 4:**
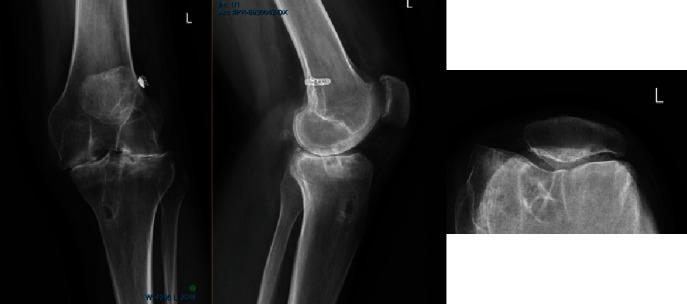
Radiograph of the left knee 7 months later in 2019.

In October 2019, Mr. BP's condition deteriorated further secondary to pain and immobility. An ultrasound guided Synvisc-One (Hylan G-F 20) injection of the left knee with 2 ml of Celestone Chronodose and 8 ml of 0.5% bupivacaine without complication. A progress radiograph (Figure 4) showed further narrowing of the medial and lateral compartments with increasing sclerosis and subarticular cysts compared to the MRI in 2018.

There continued to be a steep decline in Mr. BP's knee function and pain over the next 6 months with significant left leg wasting and reduced range of motion (10°-110°). The ACL repair also failed on examination. After consideration with another orthopaedic surgeon, a left total knee replacement (TKR) was booked. Mr. BP is now 24 years of age. Intraoperative findings were heavily haemosiderin deposition in the synovium with no active inflammation; there was complete chondral destruction consistent with severe tricompartmental arthritis. There were no intra or postoperative complications. The formal histopathological report revealed a hyperplastic synovium with a villous appearance overlying a sub synovial stroma rich in blood vessels with no polarisable foreign body, consistent with reactive synovitis with heavy haemosiderin deposition. At 6 weeks postoperatively, Mr. BP's pain as resolved, he has returned to work and has a range of motion of 0–140°.

## 2. Discussion

This case presents recurrent atraumatic haemarthrosis suspected to be secondary to an inflammatory response in the synovium to the artificial anterior cruciate ligament repair using the LARS system. The recurrent haemarthrosis eventually lead to the chondrolysis and joint destruction described above eventuating in a total knee arthroplasty at the age of 23.

Haemarthrosis associated with haemophilia has been shown to cause permanent and irreversible joint damage [1]. Haemophilic arthropathy is characterised by chronic synovitis and cartilage destruction thought to be caused by the progressive accumulation of iron as haemosiderin in the synovial membrane [1]. Iron is thought to be involved in both the synovial cell proliferation and vascular cell proliferation in the subsynovial layer resulting in an inflamed, hypervascular synovial tissue that is more susceptible to recurrent haemorrhages [3]. Furthermore, cartilage destruction is caused by the cytokines and enzymes from the inflammatory cells in the synovial membrane in combination with the increased pressure from the blood in the joint space which induces chondrocyte apoptosis of chondrocytes and in the inhibition of the synthesis of cartilage matrix [1]. In animal studies where the blood was directly injected into the legs of beagles with loading of the effected leg showed that the blood itself had a direct and harmful effects on the cartilage [2, 3].

A single atraumatic massive haemarthrosis using animal models has been shown to cause permanent and severe changes to the affected joint [4]. These changes initially start with marked joint capsule distension and blood present in local soft tissues; after 14 days, the joint capsule distension resolved; however, the haemosiderin staining persisted with evident vascular hyperplasia, and the joint space filled with dense inflammatory cell infiltrate. By 30 days, radiographical findings of affected joint showed marked cartilage and subchondral bone erosion consistent with advanced haemophilic arthropathy. As such, just one episode of haemarthrosis is able to induce the changes classically observed in advanced haemophilic arthropathy [1, 4].

### 2.1. Previous Cases

Case reports of synovitis secondary to LARS ligament are infrequent but not unprecedented. In 2012, a case report in Australia reported a large effusion with widespread synovitis and a frank haemarthrosis 12 months after a primary ACL reconstruction using a LARS artificial ligament [5]. This histopathological report in this case showed haemosiderotic synovitis in a setting of a chronic inflammatory tissue reaction to a foreign body (LARS ligament). This case was managed with a two stage revision ACL procedure with the removal of the LARS and a complete synovectomy and bone graft followed by hamstring tendon autograft 6 months later with satisfactory recovery at two weeks postoperatively [5].

A case series of 12 patients published in 2019 found that ACL reconstruction using LARS reported a high rate of synovitis (25%) among failed LARS reconstructions; however, only a singular case of synovitis with an intact LARS was noted [6]. The cause was attributed to a foreign body synovitis on histological evaluation. [6] The degree of synovitis was mild in most cases. A systematic review including 319 patients with ACL repairs with LARS reported only one case of synovitis after LARS insertion [7]. The most common reasons for failure were fixation failure at the tibial or femoral tunnel, and these rates were comparable to the traditional autograft technique [7]. While the LARS ligament has a relatively few reports of synovitis, this complication was well-recognised with the previous generations of artificial ligaments including the Leeds Keio Ligament, which was made of the same material [8, 9]. To date, this is the first case of LARS synovitis causing haemarthrosis to the degree of complete tricompartmental joint destruction resulting in total knee arthroplasty at the age of 23 years.

One of the most notable concerns was the frequent change of orthopaedic surgeon involved in the patient case. It is difficult to ascertain if the primary surgeon does not perform revision ACL reconstructions, or the decision was patient driven. Despite the four surgeons being involved, the communication between them was amicable on patient records. This was a complex and challenging case, where multiple orthopaedic surgeons where involved to guide diagnosis and treatment.

Although this is one case, a growing body of evidence of LARS induced synovitis offers a cautionary tale for the implementation of LARS ligament. Our case is an example of the potentially devastating complications of LARS induced synovitis.

## Data Availability

Case report and literature review are completed through MEDLINE and PubMed.
